# Digital Gene Expression Analysis Based on Integrated *De Novo* Transcriptome Assembly of Sweet Potato [*Ipomoea batatas* (L.) Lam.]

**DOI:** 10.1371/journal.pone.0036234

**Published:** 2012-04-27

**Authors:** Xiang Tao, Ying-Hong Gu, Hai-Yan Wang, Wen Zheng, Xiao Li, Chuan-Wu Zhao, Yi-Zheng Zhang

**Affiliations:** Key Laboratory of Bio-resources and Eco-environment, Ministry of Education, Sichuan Key Laboratory of Molecular Biology and Biotechnology, Center for Functional Genomics and Bioinformatics, College of Life Sciences, Sichuan University, Chengdu, Sichuan, People's Republic of China; Oregon State University, United States of America

## Abstract

**Background:**

Sweet potato (*Ipomoea batatas* L. [Lam.]) ranks among the top six most important food crops in the world. It is widely grown throughout the world with high and stable yield, strong adaptability, rich nutrient content, and multiple uses. However, little is known about the molecular biology of this important non-model organism due to lack of genomic resources. Hence, studies based on high-throughput sequencing technologies are needed to get a comprehensive and integrated genomic resource and better understanding of gene expression patterns in different tissues and at various developmental stages.

**Methodology/Principal Findings:**

Illumina paired-end (PE) RNA-Sequencing was performed, and generated 48.7 million of 75 bp PE reads. These reads were *de novo* assembled into 128,052 transcripts (≥100 bp), which correspond to 41.1 million base pairs, by using a combined assembly strategy. Transcripts were annotated by Blast2GO and 51,763 transcripts got BLASTX hits, in which 39,677 transcripts have GO terms and 14,117 have ECs that are associated with 147 KEGG pathways. Furthermore, transcriptome differences of seven tissues were analyzed by using Illumina digital gene expression (DGE) tag profiling and numerous differentially and specifically expressed transcripts were identified. Moreover, the expression characteristics of genes involved in viral genomes, starch metabolism and potential stress tolerance and insect resistance were also identified.

**Conclusions/Significance:**

The combined *de novo* transcriptome assembly strategy can be applied to other organisms whose reference genomes are not available. The data provided here represent the most comprehensive and integrated genomic resources for cloning and identifying genes of interest in sweet potato. Characterization of sweet potato transcriptome provides an effective tool for better understanding the molecular mechanisms of cellular processes including development of leaves and storage roots, tissue-specific gene expression, potential biotic and abiotic stress response in sweet potato.

## Introduction

Sweet potato [*Ipomoea batatas* L. (Lam.)], which belongs to the *Ipomoea* genus of the *Convolvulaceae* family, is widely grown around the world due to its strong adaptability, high and stable yield, rich nutrient content, low input requirement, easy to manage and multiple uses [1]–[3]. It has the highest energy yields per unit area per unit time among many plants, and is the sixth most important food crop in terms of production in the world [4], [5]. More than 105 million metric tons are produced globally each year, 95% of which are grown in developing countries [5]. China usually accounts for 70% and 85% of total area and yield of the world, respectively [6]. Sweet potato is a genetically challenging hexaploid (2n = 6x = 90) plant with a genome size between 2,200 to 3,000 Mbp and can hardly be considered as a model species for studies [6]–[8]. Comparing with other main crops or model organisms, the genomic resources for this crop are deficient until 2010 due to its complex hexaploid genome [8]. Therefore, genomic data sources for sweet potato were eagerly needed for gene discovery and functional studies.

High-throughput transcriptome sequencing and digital gene expression (DGE) tag profiling are efficient and economic choice for characterizing non-model organisms without a reference genome [9], [10]. Until most recently, two sweet potato transcriptomes were sequenced by the International Potato Center (CIP) and the Guangdong Academy of Agricultural Sciences of China using the Roche-454 pyrosequencing technology [11] and the Illumina/Solexa RNA-Seq technology [12], respectively. The former used leaf and stem of an African landrace, and the latter used only the roots of a new edible variety as materials. Although the former released a sweet potato gene index, it was assembled with the existing ESTs, and the sequences mainly contained short open reading frames (ORFs) [11]. The latter only delivered the original raw reads and reported the assembled scaffolds and unigenes which were assembled with SOAPdenovo [12]. At about the same time, we obtained RNA-Seq data from another sweet potato cultivar ‘Xushu 18’, which was released in 1972, but is still the leading variety in China both in annual hectarage and in total root production [13], for transcriptome studies. However, we found that there were many artifacts and defects in the assembled transcriptome data provided by the commercial assembler service. A lot of the assembled unigenes could not be read through and/or were homologous to more than one gene. Therefore, we reassembled the reads to enhance the gene accuracy and coverage, and to get a comprehensive and integrated description of the transcriptome and gene expression patterns of sweet potato.

Despite the fast development of assemblers that are able to efficiently handle more reads, transcriptome assembly is still difficult [9], [14]. The quality of a *de novo* transcriptome assembly is highly dependent on the user-defined sequence overlap length [9]. Different assemblers have different applicability and performance [15], [16]. Researchers usually chose only one assembler to assembly a transcriptome [11], [12], [17]–[19]. However, new assembly strategies such as merging the contigs of multiple assemblies [20], [21] and trimming of low-quality bases at the end of reads [22] can give better assembly results [9]. We assume that trimming of bases at the 3′-end of reads with different lengths and assembling with different assemblers, and then merging the assemblies with CAP3 can improve the *de novo* assembly.

In the present study, in order to establish a useful database of transcriptome sequence as well as of differentially expressed genes in different tissues and at different developmental stages of sweet potato, we performed *de novo* transcriptome sequencing and DGE tag profiling using the Illumina next-generation sequencing (NGS) platform Genome Analyzer II (GAII). This platform generated over 3.6 billion base pairs of DNA sequences from RNA-Seq and an average of 3.7 million tags of seven tissues from DGE sequencing. We used a combined *de novo* transcriptome assembly strategy and obtained a comprehensive and integrated transcriptomic resource with 51,736 annotated transcripts and 147 associated Kyoto Encyclopedia of Genes and Genomes (KEGG) pathways of sweet potato. Furthermore, we compared the gene expression profiles of seven tissues using DGE system and the assembled transcriptome, and identified numerous differentially and specifically expressed transcripts in different tissues and at different developmental stages of roots. This represents a fully characterized sweet potato transcriptome among tissues and developmental stages through RNA-Seq. Our data should promote the understanding of the molecular mechanisms of cellular metabolism, and it is a valuable resource for genetic and genomic studies on sweet potato in the future.

## Results

### RNA-Seq and *de novo* transcriptome assembly

To obtain comprehensive transcripts of sweet potato and an overview of its gene expression profiles in different tissues and at various developmental stages, total RNAs were isolated from seven different tissues: young leaves (YL), mature leaves (ML), stems (Stem), fibrous roots (FR) and tuberous roots at three developmental stages for RNA-Seq using the Illumina NGS platform GAII. We obtained 48,716,884 paired-end (PE) 75 bp reads corresponding to more than 3.6 billion base pairs of sequence data. Before assembly, we determined the insert size of the PE reads, and found that more than 93% of the inserts were 200 bp ±10% in length (Figure S1). We also assessed the reads quality on the Galaxy website (http://main.g2.bx.psu.edu/) [23]–[25], and trimmed the reads to form six sets of reads with 50, 55, 60, 65, 70 and 75 bp in length, respectively.

Each set of reads was *de novo* assembled into contigs with three *de novo* assemblers under optimal parameters, respectively. A total of 4,275,924 sequences (604,520,440 bp) were generated from 19 sets of contigs, including the one provided by the commercial assembler service (Table 1). Polymonomers (≥10 bp) were filtered by common perl scripts. Then, we reassembled these sequences by using CAP3 [26] to reduce redundancy and generate longer sequences, and obtained 68,227 contigs and 128,486 singletons with length ≥75 bp after the last CAP3 assembly. Sequence statistics of CAP3 assemblies is listed in Table S1. The assembled transcriptome sequences (≥200 bp) were deposited in NCBI's Transcriptome Shotgun Assembly (TSA) database under the accession numbers from JP104589 to JP160056.

**Table 1:** Statistics of *de novo* assembly output of sweet potato transcriptome.

Assembly	No. of transcripts				N50 (bp)		Average length (bp)		Total length (Mbp)		Maximal length (bp)
	> = 75 bp	> = 100 bp	> = 500 bp	> = 1,000 bp	> = 75 bp	> = 100 bp	> = 75 bp	> = 100 bp	> = 75 bp	> = 100 bp	
E75	829,797	180,039	2,675	203	77	141	94.03	154.67	78.03	27.85	1,987
E70	279,360	161,606	2,507	204	122	137	125.62	155.01	35.09	25.05	2,172
E65	240,664	142,141	2,198	172	120	137	126.62	154.90	30.47	22.02	2,802
E60	216,837	122,150	1,963	116	115	141	125.52	155.80	27.22	19.03	2,534
E55	184,687	95,969	1,596	90	111	151	124.50	159.18	22.99	15.28	2,258
E50	179,997	69,433	1,529	68	109	180	121.33	176.48	21.84	12.25	1,953
V75	162,206	130,508	8,934	1,309	236	262	199.72	226.65	32.40	29.58	3,467
V70	154,431	125,700	8,501	1,130	235	260	199.62	224.81	30.83	28.26	2,870
V65	146,690	120,234	7,628	972	227	251	197.04	220.57	28.90	26.52	2,939
V60	137,659	114,152	6,457	706	216	239	192.36	213.29	26.48	24.35	3,062
V55	126,336	105,380	4,951	453	198	219	184.85	203.41	23.35	21.43	3,274
V50	111,328	93,081	3,266	233	176	193	174.09	190.05	19.38	17.69	2,082
S75	258,392	146,435	3,885	321	143	189	140.84	184.07	36.39	26.95	2,973
S70	234,234	141,602	4,071	354	149	192	146.35	186.16	34.28	26.36	2,263
S65	222,499	135,430	4,219	361	152	195	148.23	188.48	32.98	25.53	2,363
S60	210,602	127,702	4,167	368	153	197	148.99	190.17	31.38	24.28	2,514
S55	195,912	119,012	3,919	349	154	198	149.43	190.88	29.27	22.72	2,563
S50	176,018	108,063	3,560	284	154	195	149.70	190.13	26.35	20.55	2,747
CC	208,275	154,869	8,125	1,121	194	226	177.08	207.25	36.88	32.10	3,676
FA	196,708	128,052	20,846	7,667	401	509	238.09	321.17	46.83	41.13	5,466

E75, E70, E65, E60, E55 and E50 were assembled by Edena; V75, V70, V65, V60, V55 and V50 by Velvet; S75, S70, S65, S60, S55 and S50 by SOAPdenovo. CC is contigs provided by the commercial assembler service, and FA is the final assembly with CAP3.

**Figure 1:**
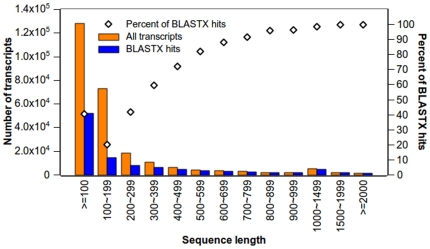
Distribution of transcripts in length and percentage of transcripts with BLASTX hits. 128,052 transcripts ≥100 bp of the final assembly were used for BLASTX search. The X-axis represents the range of the transcript length. Size distribution of the final assembled transcripts (orange) and number of transcripts with BLASTX hits (blue) shown in vertical histograms correspond to left Y-axis. The percentage of BLASTX hits to size-grouped transcripts shown in diamond corresponds to right Y-axis.

As a result, about 65% of the final assembly is ≥100 bp with an average length of 321 bp (a total of 41.13 Mbp), N50 length of 509 bp, and maximal length of 5,466 bp (Figure 1 and Table 1). There are 7,667 long transcripts that are ≥1,000 bp (Table 1) and 9,933 transcripts with ORFs ≥600 bp, 1,695 transcripts with ORFs ≥1,500 bp (Table 2). These results are obviously better than each individual assembly described above (Table 1).

**Table 2:** Summary of ORF prediction.

Assembly	Number of sequences									
	Length≥300 bp		Length≥600 bp		Length≥900 bp		Length≥1,200 bp		Length≥1,500 bp	
	Total seqs	ORFs	Total seqs	ORFs	Total seqs	ORFs	Total seqs	ORFs	Total seqs	ORFs
E75	9,704	4,852	1,524	772	332	166	82	47	16	7
E70	9,433	4,784	1,403	696	321	156	81	36	17	9
E65	8,750	4,375	1,239	601	285	133	65	37	12	10
E60	8,217	4,054	1,053	500	195	97	46	22	9	4
E55	6,976	3,409	831	364	139	72	31	16	6	3
E50	5,490	2,838	768	338	125	57	28	12	6	5
V75	25,464	13,665	5,763	3,124	1,834	1,009	682	387	262	155
V70	24,220	13,091	5,408	2,940	1,651	924	587	330	212	121
V65	22,194	12,067	4,827	2,594	1,429	767	481	254	169	89
V60	19,672	10,490	4,971	2,038	1,057	551	343	175	96	39
V55	16,016	8,444	2,933	1,455	716	355	185	88	55	24
V50	7,613	4,079	1,426	696	322	150	82	39	21	9
S75	15,724	7,348	2,228	1,138	513	289	140	67	43	31
S70	15,956	7,603	2,354	1,222	530	288	155	95	47	21
S65	15,990	7,775	2,437	1,244	561	294	147	82	50	24
S60	15,464	7,665	2,437	1,222	584	308	160	86	47	27
S55	14,674	7,310	2,348	1,171	549	303	143	69	41	21
S50	13,126	6,687	2,107	992	465	241	124	50	28	13
CC	23,826	12,940	5,219	2,909	1,654	909	564	312	208	125
UC	32,742	22,295	13,010	6,870	6,856	3,143	3,978	1,524	2,362	731
FA	37,179	25,771	16,781	9,933	9,243	5,091	5,237	2,649	2,971	1,695
SPGI	51,029	26,430	19,491	6,756	10,006	2,610	5,414	959	2,778	282

E75, E70, E65, E60, E55 and E50 were assembled by Edena; V75, V70, V65, V60, V55 and V50 by Velvet; S75, S70, S65, S60, S55 and S50 by SOAPdenovo. CC, UC are contigs and unigenes provided by the commercial assembler service. FA is the final assembly with CAP3, and SPGI is the newly published Sweet Potato Gene Index by CIP (International Potato Center).

### Quality evaluation of transcriptome assembly

**Figure 2:**
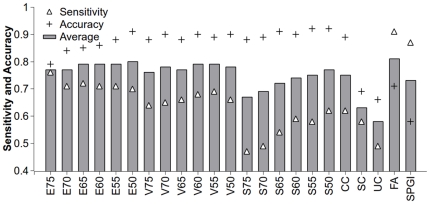
Comparison of the sensitivity and accuracy of *de novo* transcriptome assemblies. Sensitivity (triangle) and accuracy (cross) were calculated based on the result of blast (See Materials and Methods for detail) under the condition of HSPs length ≥50 bp, identity ≥90% and E-value<1e^−10^. The histogram represents the average value of sensitivity and accuracy. E75, E70, E65, E60, E55 and E50 were assembled by Edena; V75, V70, V65, V60, V55 and V50 by Velvet; S75, S70, S65, S60, S55 and S50 by SOAPdenovo. CC, SC and UC are contigs, scaffolds and unigenes provided by the commercial assembler service, respectively. FA is the final assembly, and SPGI is the newly published Sweet Potato Gene Index by CIP (International Potato Center).

To assess the quality of our assembly, local BLAST similarity search was performed using the ‘gold standard’ sequences as queries to blast against the assembled sequences. As shown in Figure 2, the average value of sensitivity and accuracy of the final assembly is higher than any of the 19 *de novo* assemblies, whereas the scaffolds and unigenes provided by the commercial assembler service have the lowest value. Taking into account the assembly statistics (Table 1), the final assembly appears to be the best among the assemblies.

Reads were also mapped back to the assembled transcripts with length ≥100 bp using Bowtie [27]. The result showed that 83.82% of the reads with seed length 50 bp mapped to 99.78% transcripts (127,769) with no more than two mismatches. According to the mapping result, the average coverage was 49.6 times, suggesting that the final assembly was highly satisfied. Then, the transcripts with length ≥100 bp were used for subsequent analysis.

We chose fourteen transcripts which have ORFs ≥900 bp and do not have BLASTX hits for new gene cloning and validation. Electrophoresis and sequence alignment results showed that all PCR products contained the predicted complete ORF except only one transcript which was slightly shorter than that expected, and the sequence identities between Sanger sequencing results and the assembled transcripts were all higher than 97% (Table S2).

### Functional annotation and classification

**Figure 3:**
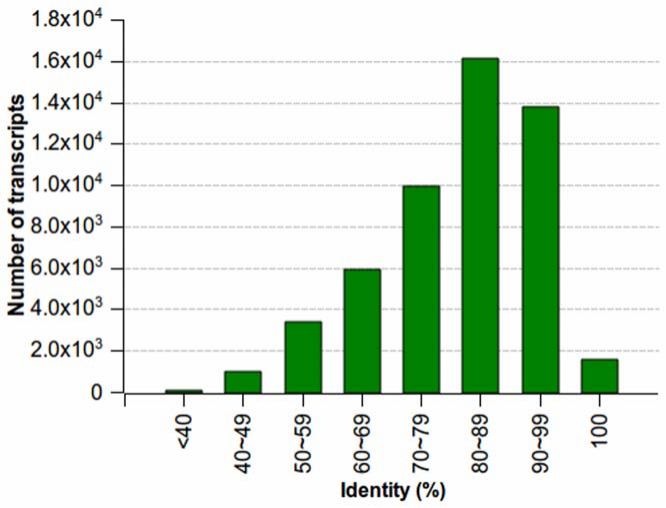
Sequence identity distribution. All BLASTX-hit transcripts were calculated. Vertical histogram shows the number of transcripts with which the range of percentage hit by BLASTX.

**Figure 4:**
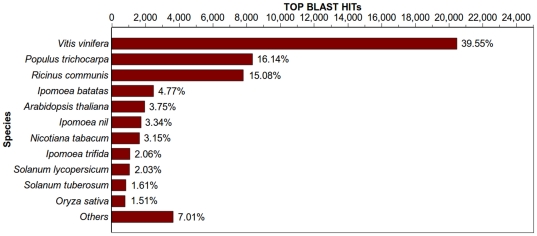
Top-Hit species distribution. 51,763 BLASTX-hit transcripts were calculated. Species with proportions of more than 1% are shown. More than 70% of the identified transcripts have the highest homology with *Vitis*, *Populus* or *Ricinus*. Less than 5% of the top matches hit sweet potato itself due to the limited number of the sweet potato protein sequences available in the NCBI database.

Sequence similarity search was conducted by Blast2GO [28]. Of the 128,052 transcripts with length ≥100 bp, 51,763 (40.42%) had significant BLASTX hits and matched to 29,056 unique protein accessions. A small number of short transcripts had BLASTX hits; while longer transcripts mostly had BLASTX hits (Figure 1). For example, less than 20% of contigs shorter than 200 bp had significant BLASTX hits, but more than 82% of contigs with length 500 bp or longer displayed significant BLASTX hits (Figure 1). Most of the BLASTX-hit sequences (41,427, 80.03%) showed sequence identity of 70% or above, and 1,556 sequences even showed sequence identity of 100% (Figure 3). Of the 29,056 unique protein accessions, 66.38% (19,288) correspond to one transcript, while 19.09% (5,546) correspond to two transcripts, and only 14.53% (4,222) to more than two transcripts. The sweet potato genes identified in this study are 18% and 40% more than previously reported by Schafleitner *et al.* [11] and Wang *et al.* [12], respectively. However, there are still a large number of sequences (76,289, 59.58%) without BLASTX hits. Most of these sequences (90%) are short fragments (≤300 bp), which may be due to the low expression levels of some genes. Some of the sequences without BLASTX hits may be new genes and/or non-coding regions. Among the 51,763 BLASTX-hit transcripts, it is worth mentioning that only 4.77% of the top matches hit sweet potato itself, which could be explained on the basis of the limited number of the sweet potato protein sequences that are currently available in the NCBI database. Most of the identified transcripts showed the highest homology with those from *Vitis vinifera* (20,472, 39.55%), *Populus trichocarpa* (8,352, 16.14%) and *Ricinus communis* (7,807, 15.08%) (Figure 4), and similar taxonomic distribution of BLASTX hits was also reported in other plant transcriptomes of asterids such as *Craterostigma plantagineum* [29] and *Fraxinus* [30].

**Figure 5:**
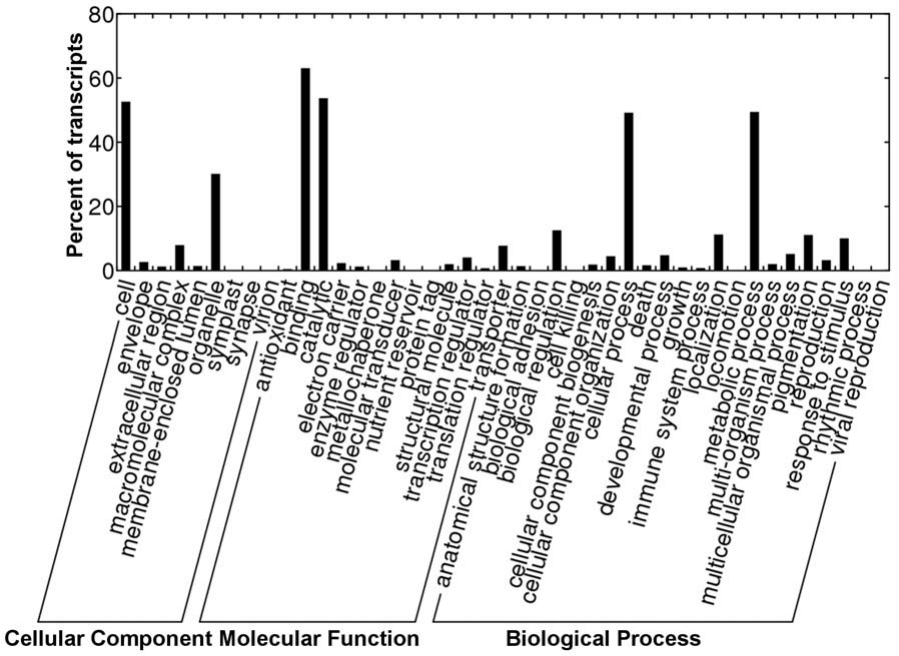
Histogram representation of GO classification. Transcripts were annotated in three categories: cellular components, molecular function and biological processes.

Gene ontology (GO) [31] assignments were used to classify the functions of the assembled transcripts. In total, we made 140,446 annotations associated with 39,677 transcripts. The terms of ‘cell and organelle’, ‘binding and catalytic’ and ‘metabolic process and cellular process’ were the most representatives of the three main categories of cellular component, molecular function and biological process, respectively (Figure 5). As expected, virion and viral reproduction were found in the categories of cellular component and biological process, respectively (Figure 5).

To survey genes involved in important pathways, annotated transcripts were mapped to the Kyoto Encyclopedia of Genes and Genomes (KEGG) pathways [32] using Blast2GO software [28]. A total of 14,117 transcripts were annotated by 17,069 Enzyme Codes (ECs), which contain 1,039 unique ECs. A total of 147 KEGG pathways were covered by 800 unique ECs in which 39 were found in the pathway of starch and sucrose metabolism (Figure S2). However, 239 unique ECs were not found in any known pathways. Sequence annotations are listed in Table S3.

### Codon usage and GC content

We analyzed the codon usage of 9,933 transcripts with ORF ≥600 bp. The results showed that the stop codon most frequently used in sweet potato is TGA, which accounted for 55.64% of all transcripts, the second one is TAA (26.73%) and TAG is the least one (17.63%). According to the codon usage analysis, we found that the most abundant amino acids encoded by the triplet codons in sweet potato are non-polar amino acids (40.7%), and then the uncharged polar amino acids (24.6%), while the acidic and basic amino acids accounted for 20.7% and 14.0%, respectively (Table S4).

Scanning ORFs of all transcripts that are ≥600 bp indicated that the GC content of sweet potato coding regions is 44.25% and AT content is 55.75%. Unlike rice and corn, whose GC distribution in the coding region has two peaks [33]–[36], the genes of sweet potato have a unimodal and narrow GC distribution (Figure S3), 52% for the first position of codon (GC1), 40% for the second (GC2) and 37% for the third (GC3), just like what were found in *Arabidopsis* and other dicot species [33].

### Identification of cDNA-derived SSR markers

We used the MISA (http://pgrc.ipk-gatersleben.de/misa/misa.html) to search for simple sequence repeats (SSRs) that are defined as dinucleotide, trinucleotide, tetranucleotide, pentanucleotide and hexanucleotide repeats at least 18 bp in length. The results showed that a total of 4,249 potential cDNA-derived SSRs (cSSRs) are distributed in 4,028 transcripts. The frequency of occurrence is 2.16% and the average distance is 11.02 kb in sweet potato transcriptome sequences. Most of these cSSRs (31.14%) are trinucleotide repeats, followed by hexanucleotide repeats (29.23%) and dinucleotide repeats (26.55%), and only a small portion of them are tetranucleotide and pentanucleotide repeats (5.86% and 7.23%), respectively. There are 181 transcripts containing more than 1 cSSR, and 129 cSSRs represent in compound formation. Among all cSSRs, the motif AG/CT has the highest frequency (18.10%), followed by motifs AAG/CTT (11.11%) and AT/AT (6.02%). In total, there were 338 unique motifs. The cSSRs identified in this study are valuable resource for genetic analysis of sweet potato.

### Statistics of DGE tags

To characterize the digital gene expression profiles in sweet potato, seven DGE libraries were constructed and sequenced using Illumina deep sequencing technology. More than 3.5 million raw tags were obtained in each library (Table 3). About 95% of the raw tags passed the filter, resulting in 24,557,853 clean tags in which there were 328,383 distinct clean tags in total. The clean tags in each sample ranged from 3.35 to 3.63 million, and the distinct clean tags ranged from 93,593 to 139,389 (Table 3). The clean tags data are deposited in NCBI's Gene Expression Omnibus [37] and are accessible through GEO Series accession number GSE35929 (http://www.ncbi.nlm.nih.gov/geo/query/acc.cgi?acc=GSE35929).

**Table 3:** Statistics of DGE library sequencing and tag mapping.

Summary	YL	ML	Stem	FR	ITR	ETR	HTR	Averages
Raw Tags	3,536,026	3,630,797	3,616,244	3,847,265	3,823,315	3,711,657	3,745,496	3,701,543
Clean Tags	3,352,753	3,429,018	3,453,654	3,583,907	3,630,619	3,566,630	3,541,272	3,508,265
Clean Tags/Raw Tags (%)	94.82	94.44	95.50	93.15	94.96	96.09	94.55	94.79
Distinct Raw Tags	307,858	302,173	280,858	384,868	280,755	237,874	299,486	299,125
Distinct Clean Tags	125,145	118,397	118,715	139,389	109,697	93,593	115,636	117,225
Distinct Clean Tags/Clean Tags (%)	3.73	3.45	3.44	3.89	3.02	2.62	3.27	3.35
Mapped Clean Tags	2,506,655	2,894,248	2,904,513	2,890,484	3,177,381	3,101,355	2,764,216	2,891,265
Mapped Clean Tags* (%)	74.76	84.40	84.10	80.65	87.52	86.95	78.06	82.35
Mapped Distinct Clean Tags	69,118	76,860	75,256	81,687	74,598	60,619	68,341	72,354
Mapped Distinct Clean Tags * (%)	55.23	64.92	63.39	58.60	68.00	64.77	59.10	62.00
UCT Mapping to Transcript	2,154,554	2,529,417	2,551,795	2,504,573	2,760,652	2,699,212	2,363,845	2,509,150
UCT Mapping to Transcript* (%)	64.26	73.77	73.89	69.88	76.04	75.68	66.75	71.47
UDCT Mapping to Transcript	51,109	56,449	57,232	59,107	54,378	43,755	49,798	53,118
UDCT Mapping to Transcript* (%)	40.84	47.68	48.21	42.40	49.57	46.75	43.06	45.50
Tag-mapped Transcripts	30,977	32,131	33,754	35,109	32,900	28,645	30,730	32,035
Unambiguous Tag-mapped Transcripts	25,558	26,717	28,013	29,162	27,244	23,490	25,309	26,499
Unknown Tags	846,098	534,770	549,141	693,423	453,238	465,275	777,056	617,000
Unknown Tags* (%)	25.24	15.60	15.90	19.35	12.48	13.05	21.94	17.65
Distinct Unknown Tags	56,027	41,537	43,459	57,702	35,099	32,974	47,295	44,870
Distinct Unknown Tags* (%)	44.77	35.08	36.61	41.40	32.00	35.23	40.90	38.00

* : Percent of Clean Tag; UCT: Unambiguous Clean Tags; UDCT: Unambiguous Distinct Clean Tags; YL: Young leaves; ML: Mature leaves; Stem: Stems; FR: Fibrous roots; ITR: initial tuberous roots; ETR: expanding tuberous roots; HTR: harvest tuberous roots.

When clean tags were mapped to the final assembled transcripts described above, it was found that the clean tags mapped to transcripts in each sample ranged from 2.50 to 3.18 million, accounting for 74.76% to 87.52% (Table 3). The distinct clean tags mapped to transcripts ranged from 60,619 to 81,687 in different samples, accounting for 55.23% to 68.00% (Table 3). A total of 48,600 transcripts could be mapped by the clean tags. Of the seven libraries, the number of tag-mapped transcripts ranged from 28,645 to 35,109 (Table 3). The digital gene expression data are listed in Table S3.

### Analysis of differential gene expression

**Figure 6:**
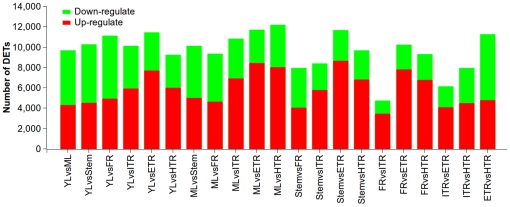
Transcripts differentially expressed between different tissues. Up-(red) and down-regulated (green) transcripts were quantified. The results of 21 comparisons between each two samples are shown.

To compare differential expression patterns among seven libraries, we normalized tag distribution for gene expression level in each library to make an effective library size and extracted significance of differentially expressed transcripts (DETs) with *p* value≤0.05 and log2 fold-change ≥1 by edgeR (Empirical analysis of Digital Gene Expression in R) [38], which provides an empirical approach and eliminates a bias being introduced by RNA composition [39], according to the user manual. We compared these seven libraries pair-wisely so that 21 pairs of comparison were implemented. Among these comparisons, we found that 4,721 to 12,151 transcripts had significant changes in expression, and the average number was 9,657 (Table S3). The up-regulated and down-regulated transcripts are shown in Figure 6. The differential expression patterns among libraries revealed that the largest differences occurred between ML and HTR (harvest tuberous roots), and there were 7,994 and 4,157 transcripts up- and down-regulated in ML, respectively. The top five up-regulated transcripts in ML are non-photosynthetic ferredoxin, thiamine biosynthetic enzyme, glycolate oxidase, ribulose bisphosphate carboxylase, chloroplast LHCII type I chlorophyll *a*/*b* binding protein (Cab). The top five up-regulated transcripts in HTR are sporamin A, sporamin B, expansin 2, sucrose-phosphate synthase, aspartyl protease. The smallest difference was shown between FR and ITR (initial tuberous roots), in which only 4,721 DETs were identified. The differences of other comparisons ranged from 6,109 to 11,664 transcripts.

**Figure 7:**
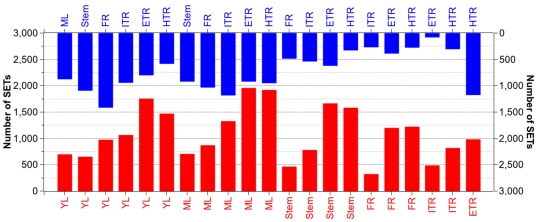
Transcripts specifically expressed between different tissues. Specifically expressed transcripts were quantified. The numbers of DGE transcripts of 21 comparisons between each two samples are shown in blue and red histograms (top vs. bottom).

We also observed a large number of specifically expressed transcripts (SETs) between each two libraries (Table S3). SETs were defined as those did not express in one library but the tag numbers were larger than 11 in another one. Comparisons showed that there were 559 to 2,874 SETs with an average number of 1,808 among 21 comparisons (Figure 7). The largest difference was observed between ML and ETR (expanding tuberous roots) and there were 1,952 and 922 transcripts specifically expressed in ML and ETR, respectively. The smallest difference was seen between ITR and ETR, in which only 477 and 82 transcripts were specifically expressed, respectively.

All DETs were used for function enrichment analysis by R tools. For pathway enrichment analysis, we mapped those DETs to terms in KEGG database and searched for KEGG terms that were significantly enriched comparing with the transcriptome background. We applied hypergeometric test and adjusted the *p*-value using Bonferroni method [40] to identify significantly enriched pathways. Results showed that the functional transcripts involved in ‘porphyrin and chlorophyll metabolism’ pathway were enriched in ML as compared to other six libraries. Comparison between ETR and HTR displayed that transcripts involved in ‘carbon fixation in photosynthetic organisms’ pathway were enriched in ML. When comparing to all roots libraries (FR, ITR, ETR and HTR), transcripts being active in ‘polyketide sugar unit biosynthesis’ pathway were enriched in Stem. Furthermore, all SETs were also used for functional enrichment analysis. When comparing all root libraries with YL and ML, the SETs involved in ‘carbon fixation’ pathway were enriched in leaves. The results also demonstrated that during tuberous root development, the SETs involved in ‘starch and sucrose metabolism’ pathway were enriched in ETR comparing with ITR.

In order to study the tissue-specifically expressed transcripts, we treated young and mature leaves (named leaves), initial, expanding and harvest tuberous roots (named tuberous roots), fibrous and tuberous roots (named roots) as biological replicates, respectively. Comparisons displayed that there were 2,139 differentially expressed transcripts between leaves and roots, in which 2,001 transcripts were up-regulated in leaves and 138 transcripts up-regulated in roots, including 835 transcripts specifically expressed in leaves and 119 ones in roots (Table S3). Significance enrichment was observed in ‘carbon fixation in photosynthetic organisms’, ‘glyoxylate and dicarboxylate metabolism’, ‘porphyrin and chlorophyll metabolism’ pathways in both differential and specific expression. Transcripts involved in photosynthesis were observed to be differentially expressed, including genes encoding ribulose bisphosphate carboxylase, cab-like protein, photosystem Ι reaction center subunit chloroplast and proton gradient regulation 5. All these genes were up-regulated in leaves and are functional essential for carbon dioxide assimilation. Expression patterns of some stress-responsive or defense-related genes were also differentially expressed, such as those encoding cdsp32 protein, cristal-glass1 protein, ripening protein and phytochelatin synthetase-like protein.

### Gene expression during root development and carbohydrates accumulation

To better understand gene expression during root development and carbohydrate accumulation, we collected the FR, ITR, ETR and HTR at 1, 1.5, 3 and 5 months after planting, respectively. The ITR and ETR were sampled during the early stages of storage root formation, while the HTR was sampled at the late stage. The DGE results (Table S3) showed that ITR and ETR had the highest expression levels for UDP-glucose pyrophosphorylase. The expression level of sucrose synthase in ITR was 4,066 TPM (number of transcripts per million clean tags), but this value for FR, ETR and HTR was only 1,938, 1,652 and 1,101, respectively. Sucrose-phosphate synthase was up-regulated in tuberous roots comparing with FR, and highly expressed in ETR and ITR. These three enzymes play important roles in sucrose biosynthesis pathway, and their expression patterns match well with early studies [41], [42]. For ADP-glucose pyrophosphorylase (AGPase), which is involved in starch accumulation, HTR and ETR had the highest expression levels. Invertase inhibitor-like protein and fructokinase were both elevated in tuberous roots comparing with FR. The expression of granule-bound starch synthase I, sporamin A and sporamin B was all elevated in rapid bulking periods. In addition, the differential expression of 19 out of 22 genes possibly related to storage root induction identified by You *et al.* [43] was observed in our study.

Dozens of genes involved in storage root induction were found to be differentially expressed, including those in cell division, regulation of transcription, membrane transport, and stress response. Class III HD-Zip protein 8 was elevated in FR and was reported to play a role in regulating the development of cambia and secondary vascular tissues [44]. Differential expression was observed for short-root protein, which is a key regulator in root radial patterning, meristem maintenance [45] and asymmetric cell division [46], and this gene was highly expressed in FR. The expression of Spf1 protein, a regulator for sporamin and beta-amylase gene [47] displayed the same pattern as short-root protein.

### Candidate genes for potential abiotic stress tolerance and insect resistance

Among the genes annotated in the sweet potato transcriptome, we found that there were a large number of genes that could respond to drought, salt, cold, heat or osmotic stresses (Table S3). Such as those encoding metallothionein (MT), Mn-superoxide dismutase (MnSOD), catalase (CAT), vacuolar H^+^-pyrophosphatase (PPase), ascorbate peroxidase (APX), polyphenol oxidase (PPO), late embryogenesis abundant proteins (LEA), Na^+^/H^+^ antiporter (NHX), early-responsive to dehydration stress protein (ERD), aquaporin (AQP), vacuolar cation/proton exchanger (CAX), betaine aldehyde dehydrogenase (BADH), and abscisic acid responsive elements-binding factor (AREB). The DGE analysis showed that most of these genes had relatively high expression levels. The highest expression level in seven tissues was 7,790 TPM for MT, 7,734 for MnSOD, 5,990 for CAT, 2,460 for PPase, 1,903 for APX, 1,671 for PPO and 1,360 for ethylene-responsive element binding protein (EREBP) genes.

Plant proteinase inhibitors (PIs) are toxic to insect pests [48]. Genes coding for several PIs were found in this study, such as kunitz-type protease inhibitor, cysteine protease inhibitor, trypsin inhibitor. These genes displayed different expression patterns (Table S3). For example, at least four different cysteine protease inhibitor genes were found in DGE libraries, two of them were highly expressed in ETR (987 and 94), while the other two were highly expressed in ML (1,041) and Stem (1,194), respectively. Sporamins, which are the major storage proteins in sweet potato tuberous roots, belong to kunitz-type protease inhibitors and can be grouped into two subfamilies [49], [50], were abundant in some tissues, but very pool in other tissues. Sporamin B had the highest expression level in HTR (4,213), while almost none in YL (0), ML (1) and Stem (0). For one sporamin A, the highest expression level in HTR reached 110,994, while only 5 in YL. However, another sporamin A showed the highest expression level in YL (4,486), and extremely low in other tissues, only 26 for ITR, 17 for ML, 4 for Stem, and not expressed in FR, ETR and HTR. Some sporamin A genes were also expressed in all the seven tissues. Therefore, the existence and differential expression patterns of these potential abiotic and biotic stress genes in sweet potato may be an important reason for sweet potato's strong adaptation capacity in nature.

## Discussion

### Improving *de novo* transcriptome assembly

In published papers, authors usually employed one commonly used software to construct transcriptomes with short reads data. In this study, we used a combined strategy to conduct *de novo* assembly of sweet potato transcriptome on the basis of Illumina/Solexa short reads. We trimmed the bases at the 3′-ends of reads in different lengths (5–25 mers at 5 mers interval) to obtain six read sets. Each of them was assembled under various parameters by using different *de novo* assemblers. These assemblers implement different assembly algorithms including the traditional OLC approach (Edena) [51] and the de Bruijn graph approach (Velvet and SOAPdenovo) [52], [53]. The results showed that, Velvet produced better assembly result than SOAPdenovo, while Edena generated more short contigs (Table 1). Different assemblies resulted in different N50 values. When 75 bp reads were assembled by using Edena, Velvet and SOAPdenovo, the N50s (contig ≥100 bp) were 141, 262 and 189 bp, respectively. However, trimmed reads decreased the N50 of Velvet assembly, but increased the N50 of Edena and SOAPdenovo (Table 1). All the contigs in individual assembly including the one assembled by commercial assembler service were still too short to be used, and some overlaps still existed between the contigs. Therefore, merging of them could get better results. Hence, we clustered the contigs of the 19 assemblies by CAP3 [26]. The results displayed that the final assembly was markedly improved in contiguity in terms of N50, average, maximal and total sequence length, and number of long sequences as compared to each single assembly (Table 1). The final assembly output was also much better than the recent study on sweet potato root transcriptome, which was assembled with the SOAPdenovo software [53]. Although ours had 20% less reads than theirs, our assembly has higher contiguity, i.e. with N50 length 401 bp versus 252 bp, average contig length 238 bp versus 202 bp and number of contigs (>1,000 bp) 7,650 versus 3,024 [12]. We also carried out scaffolding the final assembly by using SSPACE [54], but only a small portion of the transcripts could be merged (data not shown). Therefore, we suggest that trimming of all raw reads sequences at the 3′-ends and merging of assemblies from different assemblers could significantly improve the assembly outcome.

### Evaluation of *de novo* transcriptome assembly quality

There were no standard criteria to evaluate the quality of transcriptome assemblies [20]. Researchers assess the quality of an assembly mostly by looking at the contiguity and accuracy of the assembly [55]. In addition to the contiguity, we also assessed the assembly quality with several metrics. Due to lack of genomic resources for sweet potato, the sweet potato mRNAs with full-length cds from GenBank were considered as ‘gold standard’ reference in our studies. To calculate the sensitivity, the overlapped high-scoring segment pairs (HSPs) were only calculated once. We also considered the effect of the e-value threshold on sensitivity calculation, but there seems to be little effect (data not shown). For each individual assembly, Edena achieved higher sensitivities than Velvet and SOAPdenovo. But the final assembly produced by CAP3 had the highest sensitivity (Figure 2). To calculate the accuracy, all unmatched parts were considered as false positives. As the terminal sequences usually could not match the alignments, we considered the effect of terminal length on accuracy calculation. But the terminal length seems to have little effect (data not shown). As there is usually a trade-off between contiguity and accuracy [55], we considered the average value of sensitivity and accuracy as the metric. The final assembly's average value of sensitivity and accuracy was higher than any single assembly as well as the newly published sweet potato gene index (SPGI) [11] (Figure 2). The accuracy of the final assembly may be underestimated, because many genes may exist as gene families, and the untranslated regions (UTRs) are relatively less conserved.

Moreover, graphical visualization tools such as Tablet [56] may be very valuable for assessing assembly quality [55]. Mapping the reads to the final assembly using Bowtie [27] allows us to visualize the assembly quality.

Furthermore, we evaluated the ORFs of assembled sequences. The final assembly had more transcripts with longer ORFs than any single assembly and the recently released SPGI [11] (Table 2). All results from above metrics indicate that our final assembly quality is the most satisfying.

Due to lack of genome resources, DGE tags were mapped to the assembled transcriptome for gene expression analysis and 74.76% to 87.52% of the clean tags in the seven libraries mapped to the transcriptome sequences. While the clean tags were mapped to the 75 bp reads, 87.46% to 95.85% of tags hit. But only 28.91 to 34.74% clean tags mapped to the unigenes provided by the commercial assembler service, and 69.68% to 79.83% to the previous reported SPGI [11]. This also indicates that our final assembled transcriptome is more comprehensive and integrated.

### Comparison of the combined assembly with Trinity assembly

Our combined assembly was finished in September 2010. In the preparing of this paper, a new *de novo* transcriptome assembly package “Trinity” was developed at the Broad Institute [57]. As Trinity seems to be the standard now, we reassembled our data using Trinity (release 2011-11-26) and compared the results obtained from these two assembly strategies. The Trinity assembling is more user-friendly and time-saving as compared to our combined strategy. However, we found that our previous assembled results were more preferable based on deeply comparison and analysis (Table S5).

First, Trinity produced much more transcripts with length >200 bp, and a little more long transcripts (>1 kb) than the combined assembly. But the combined assembly generated better results in mean length, maximal contig length and N50. It is worth noting that 67,610 transcripts (>200 bp) from the Trinity assembly were derived from 50,609 components (genes). But the combined assembly produced 55,181 contigs with length >200 bp. The result of the predicted ORFs using EMBOSS [58] demonstrated that for transcripts ≥300 bp, the Trinity assembly had more transcripts while the combined assembly had higher percentage and more transcripts of long-ORFs (≥900 bp).

Second, we mapped the reads back to these two assemblies using Bowtie [27] provided in the Trinity package with default parameters. The results showed that 57.96% and 60.58% reads were mapped back to the Trinity assembly and the combined assembly, respectively. Moreover, we compared the DGE mapping results and found that more tags could mapped to the combined assembly than the Trinity assembly in all samples except sample YL.

Third, comparison of the Trinity assembly with the combined assembly using BLAT (version 34) [59] indicated that the shared transcripts accounted for around 95% of the total number of transcripts, and most of the unique (none shared) sequences were short fragments. This demonstrates that most of the sweet potato transcriptome sequences could be reconstructed by either the combined strategy or the Trinity approach. In addition, the top BLASTX-hits indicated that most of the identified transcripts of these two assemblies had the highest homology with *Vitis*, *Populus* or *Ricinus*. This indicates that these two assemblies are somehow equivalent in the reconstruction of total number of genes.

Fourth, evaluating the accuracy and sensitivity using full-length cds of sweet potato from GenBank as reference showed that Trinity assembly had a little higher accuracy (0.73 *vs.* 0.72), but lower sensitivity (0.79 *vs.* 0.85) than the combined assembly.

Similar results were also reported in a recently published paper [60]. Zhao *et al.* compared several *de novo* transcriptome assemblers and different assembly strategies, and found that Trinity was the best single *k-mer* assembler for transcriptome assembly [60]. However, in order to achieve a better assembly, multiple *k-mer* approach should be considered [60]. In our combined assembly strategy, we have not only used multiple *k-mer* method but also different assemblers.

### Global gene expression patterns of sweet potato

The transcriptome differences of the seven tissues (including roots at different developmental stages) were characterized using Illumina DGE technology, which is essentially an improved version of MPSS [61], [62]. Comparing with other genome-wide microarray expression profiling platforms, deep sequencing-based expression analysis does not need laborious and costly cloning and sequencing steps [62]. It gives an unbiased methodology to investigate expression pattern for each gene based on digital signal and does not depend on reference genome. DGE can eliminate background signals caused by cross-hybridization and share a higher consistence with qPCR than other platforms [62]. Being superior, NGS has been widely used since its inception [63]–[68]. A large number of short reads generated by NGS systems require efficient development of algorithm and software in computing. Some differential expression analysis tools have been developed and widely used in the last several years, some of which were developed for deep sequencing technology, such as DESeq [69], DEGseq [70] and edgeR [38]. In this study, edgeR was chosen because it could be used for RNA-Seq, Tag-Seq and SAGE experiments, and it describes an empirical approach to estimate the bias introduced by RNA composition and integrates that into the effective library size.

We compared the gene expression variations between each two libraries, and identified numerous differentially expressed transcripts between tissues and different developmental stages (Table S3). To verify whether these DETs identified by DGE were reliable, we compared the results with those from previous studies. You *et al.* [43] constructed a cDNA library and identified 22 genes differentially expressed between fibrous and tuberous roots. In our study, 19 of these genes (86.4%) were found to be differentially expressed and only the J8-like protein, NAM-like protein, and G10-like protein had no differential expression. Actually, there is no CATG site in the transcripts encoding J8-like protein and NAM-like protein. Only the transcript encoding G10-like protein showed the same expression levels in fibrous and tuberous roots. When analyzing the expression between leaves and roots, the specifically expressed transcripts involved in carbon fixation and photosynthesis were all up-regulated or specifically expressed in leaves, while some sporamins and starch-related genes were up-regulated in roots. These all illustrate that gene expression profiling from DGE can give reliable results.

We also found a large number of potential stress tolerance and insect resistance related genes in sweet potato. Most of these genes were first reported in this crop, such as SIZ1, PPase, CAX, ERD, LEA and AQP. The expression patterns of these genes were characterized. For example, the PPO gene was highly expressed in YL, CAT in ML, APX in FR, BADH in ITR, MnSOD in ETR and MT in HTR (Table S3). Several PIs that may play important roles in insect pest resistance were found too, and displayed high expression levels in some tissues (Table S3). Sporamins, which account for 60% to 80% of the total soluble proteins in sweet potato tuberous roots, belong to the PIs and are considered to be tuber-specific [49], [50]. Usually the expression levels of sporamins are very high. But in this study, we found that not all sporamin genes were specifically expressed in roots and high level of expression also existed in leaves. These may help us to explain why sweet potato has such a strong adaptation capability and can produce tuberous roots with a little irrigation and pesticide.

### Discovery of viruses with RNA-Seq

NGS makes virus identification easier. With the application of Life Sciences 454 high-throughput sequencing, viruses could be identified in genomes of animals [71] and plants [72]. Kreuze *et al.* [73] identified some novel viruses and the sequence of an entire viral genome by the Illumina's small RNA sequencing technology. Coetzee *et al.* [74] used the sequencing-by-synthesis technology offered by the Illumina Genome Analyzer II to characterize the virome of a vineyard. These approaches could not only identify known viral pathogens that occur at extremely low titres, but also novel viruses, without the necessity of any prior knowledge. Moreover, these methodologies are able to detect both RNA and DNA viruses.

Sweet potato viral diseases are the major reasons for yield loss and cultivar decline and cause over 20% of yield losses in China, with the most severe case reaching 78% reported in Shandong province [6], [75]. Using RNA-Seq, we also detected both RNA and DNA viruses, and novel viruses. Among the BLASTX-hit transcripts, there were 79 virus sequences putatively belonging to 12 viral species (Table S3). Two of them were DNA viruses, and were previously reported only in Peru [73] and Tanzania [76]. Surprisingly, four viruses were first found in China, and some viruses were first found in sweet potato. Due to lack of genome sequences of some viruses, although 77 out of 79 putative viral transcripts were found to have BLASTX-hit sequence identity >75%, some of them may still need to be confirmed. More sequence information for these viruses is needed. Five viruses with 100% homologies of BLASTX-hit to known viruses are *Sweet potato feathery mottle virus* (SPFMV), *Sweet potato virus G* (SPVG), *Sweet potato latent virus* (SwPLV), Ipomoea vein mosaic virus (IVMV) and Mikania micrantha mosaic virus (MMWV). Most viral transcripts belong to SPFMV which is the most common sweet potato virus worldwide [77]. MMWV was only reported in *Mikania micrantha* H.B.K. in Guangdong province, China [78]. During the last decade, 11 viruses were reported in China [75], but to our knowledge, this is the first time to detect so many viruses in one sweet potato variety from one field.

It was estimated that sweet potato viral diseases caused annually economic losses of four billion Chinese Yuan to the sweet potato industry in China [75]. But how the viruses cause the yield loss is unknown. Previous studies revealed that vegetative growth and physiology of virus-infected sweet potato plants contributed to losses of roots yield [75]. Virus-free plants showed much better vegetative growth than virus-infected ones and roots of virus-free plants developed earlier and expanded faster at early stage of root development than those of virus-infected ones [75]. Based on DGE analysis, we found that the gene expression levels of the viral genes were extremely low in leaves. However, the viral genes of SPFMV, SPVG and SwPLV were highly expressed in FR and tuberous roots, while those of IVMV were highly expressed in ETR (Table S3). Depending on the number of FR forming storage roots, sweet potato plants yield either a high number of uniform and high-grade roots, or a low number of large storage roots per plant or no marketable roots at all [79]. Well-developed root system is beneficial for water uptake and nutrient absorption, and their transport to sink organs, thus resulting in increased root yield [75]. We, therefore, believe that the high level of viral gene expression in roots, especially in fibrous roots may affects the function and development of the root system (e.g. uptake of water and nutrients, formation and expanding of tuberous roots), eventually leads to yield loss and cultivar decline.

## Materials and Methods

### Plant material and RNA extraction

Stem cuts of sweet potato [*I. Batatas* (L.) Lam. cv. Xushu 18] were planted in June, 2009, and grown under normal conditions in Chengdu, Sichuan Province of China. Tissue samples of young leaves (YL), mature leaves (ML) and stems (Stem) were collected at 1.5 months after planting. Fibrous roots (FR), initial tuberous roots (ITR), expanding tuberous roots (ETR) and harvest tuberous roots (HTR) were collected at 1, 1.5, 3 and 5 months after planting, respectively. All tissue samples collected were snap-frozen immediately in nitrogen and stored at −80°C until further processing.

Total RNAs were extracted using the Trizol® Reagent (Invitrogen, USA), and treated with DNase I (Fermentas, USA) according to the manufacturer's instructions. RNA quality and purity were assessed with OD_260/230_ ratio and RNA integrity number (RIN) by using the SMA3000 and the Agilent 2100 Bioanalyzer, respectively.

### Transcriptome sequencing

To obtain a comprehensive list of transcripts, equal amounts of total RNA from each sample were pooled together. The poly (A)^+^ RNAs were purified from the mixed high quality total RNAs (20 µg) on oligo(dT) Dynabeads and impurities were removed from the hybridized sample with a series low-salt solution washes. The purified poly (A)^+^ RNAs were then dissolved into a Tris-based buffer, precipitated with 70% ethanol, and then resolubilized. First strand cDNAs were synthesized using Oligo(dT) primer, then second strand cDNAs were synthesized using RNase H and DNA polymerase I. Double stranded cDNAs were random fragmented using Nebulizer, then repaired and added an adenine base to the 3′ end. Two different adapters were ligated to 5′ and 3′ ends, respectively. The ligated fragments were separated on gel, and the fragments about 200 bp were extracted. After amplification by PCR, the fragments were separated using electrophoresis and purified, then submitted to Illumina GA II platform for sequencing at Beijing Genomics Institute (BGI)-Shenzhen, Shenzhen, China (http://www.genomics.cn). Raw sequence data were generated by Illumina pipeline and were available in NCBI's Short Read Archive (SRA) database (http://www.ncbi.nlm.nih.gov/Traces/sra/sra.cgi?) under accession number SRA043582.

### *De novo* transcriptome assembly and evaluation

The pipeline of *de novo* transcriptome assembly and analysis is shown in Figure S4. All the assemblies were run on a 64-bit Linux system (Ubuntu 10.10) with 32G physical memory except those assembled by BGI (referred as commercial assembler service in above description). Reads quality was assessed on the Galaxy website (http://main.g2.bx.psu.edu/) [23]–[25]. As most of the RNA-Seq experiments, reads quality at the 3′ end dropped down. The median Phred quality score was below 30 (which means the sequencing error was more than 0.1%) from cycle 61 and 56 of the forward and the reverse reads, respectively. So, the 3′ ends of the clean reads were trimmed, ranging from 5 to 25 mers at 5 mer intervals, to form 6 sets of reads, which were used for assembly with *de novo* assemblers of Edena v2.1.1 [51], Velvet v1.0.12 [52] and SOAPdenovo v1.04 [53] using different parameters, respectively. Statistics data of 330 assemblies were generated according to the assembled contigs by common perl scripts (data not shown). All the best assemblies obtained from each set of reads by using every assembler with the optimized parameter were pooled, together with the contigs provided by the commercial assembler service (19 sets of contigs in total), and then were reassembled with CAP3 [26]. The raw reads were also assembled using Trinity release_2011-11-26 [57] with default parameters.

In addition to the statistics of each assembly, another method was used to evaluate the quality of the assemblies. The known mRNA sequences with full-length cds of sweet potato available in GenBank were considered as ‘gold standard’ reference in this study, and used to blast against each assembly by BLASTN [80]. Based on the blast results, we considered the average of sensitivity and accuracy of each assembly. Sensitivity, also known as integrity or transcriptome coverage, is the ratio of the sum of all unique aligned segment length to the reference length. We calculated the sensitivity with *Sen* = TP/(TP+FN) (TP = true positives, FN = false negatives), where *Sen* is sensitivity, TP is the sum of all aligned segment length (the overlap aligned regions were only calculated once), FN is the sum of all reference segment length that were not aligned. Accuracy is the ratio of the sum of all unique aligned segment length to the assembled transcript length. We calculated the accuracy with *Acc* = TP/(TP+FP) (FP = false positives), where *Acc* is accuracy, FP is the sum of all assembled segment length that were not aligned.

To further assess and/or visualize the assembly, we mapped the reads onto the final assembled transcripts using the Bowtie program [27] available at the Galaxy website (http://main.g2.bx.psu.edu/) [23]–[25]. The results were viewed by Tablet [56].

Moreover, we evaluated the assemblies by scanning ORF with EMBOSS package [58]. Fourteen transcripts with long ORFs but without BLASTX hits were selected for reverse transcription-polymerase chain reaction (RT-PCR) validation. Primers were designed according to assembled transcripts using Primer Premier 5.0 (PREMIER Biosoft. International, CA, USA) and sequence amplifying was implemented using KOD-Plus-Neo DNA polymerase (Toyobo, Japan). PCR products were sequenced by Sanger method.

### Functional annotation and classification

The final assembled transcripts (≥100 bp) were submitted for homology and annotation searches using Blast2GO software v2.4.4 [28]. For BLASTX against the NR database, the threshold was set to E-value≤10^−6^. GO classification was achieved using WEGO software [81]. Enzyme codes were extracted and Kyoto Encyclopedia of Genes and Genomes (KEGG) [32] pathways were retrieved from KEGG web server (http://www.genome.jp/kegg/).

### Sequence analysis

After sequence assembly, we extracted ORFs using EMBOSS [58], and analyzed GC content and codon usage bias using DNAStar (http://www.dnastar.com/) and CodonW (http://codonw.sourceforge.net/). We also searched cDNA-derived simple sequence repeats (SSRs) using a perl script known as MIcroSAtellite identification tool (MISA, http://pgrc.ipk-gatersleben.de/misa/misa.html).

### DGE library preparation and sequencing

Total RNAs were extracted from the seven tissues individually, and their qualities were assessed as described above. The poly (A)^+^ RNAs were purified from 6 µg of total RNAs, and cDNAs were synthesized as described above for each sample. The double stranded cDNAs were digested with *Nla* III to produce a CATG cohesive end, followed by purification with Dynabeads and ligation to Illumina adapter I that contains a *Mme* I restriction site. cDNA fragments containing adapter I were purified and digested with *Mme* I which recognizes the junction of the adapter I and the CATG site, and makes a cut at 17 bp downstream of the *Nla*III recognition site. The 21 bp tags containing adapter I were ligated to Illumina adapter II to generate a tag library. These tag fragments were amplified by liner PCR for 15 cycles using PCR-primers annealed to the adapter ends. The 85 bp amplicons were separated on 6% TBE PAGE gel, purified and denatured to produce single strand molecules. These molecules were anchored to Solexa sequencing array and sequenced on Illumina GA II at BGI-Shenzhen, Shenzhen, China (http://www.genomics.cn). Raw sequence data were generated by Illumina pipeline and were available in NCBI's SRA database (http://www.ncbi.nlm.nih.gov/Traces/sra/sra.cgi?) under accession number SRA043583.

### Analysis of DGE tags and mapping to reference transcripts

Pipeline for DGE analysis was shown in Figure S4. The 21 bp DGE tags were extracted, filtered and counted by custom shell scripts. Raw sequences were transformed into clean tags as described [82]. All clean tags were aligned to the final assembled transcripts using Bowtie [27] available at the Galaxy website (http://main.g2.bx.psu.edu/) [23]–[25], allowing no more than one nucleotide mismatch. In order to compare the expression abundance among samples, tags were normalized to TPM (number of transcripts per million clean tags) [62], [63].

### Analysis of differential expression

The edgeR package [38] was used for differential expression analysis of genes. We compared each two libraries and used hypergeometric test to identify differentially expressed transcripts (DETs), specifically expressed transcripts (SETs) and functionally enriched transcripts.

## Supporting Information

Figure S1 **The insert size histogram of PE reads.** The insert size of PE reads was inferred by mapping the PE reads to a chloroplast genome of *I. purpurea* (GenBank Accession Number NC_009808) which is a species close to *I. batatas*.
(TIF)

Figure S2 **Map for KEGG starch and sucrose metabolism pathway of sweet potato.** ECs in red were found in this study.
(TIF)

Figure S3 **Distribution of GC in the coding region of sweet potato.** 9,933 transcripts with ORF≥600 bp were used for GC content analysis. GC: GC contents of entire ORFs; GC1, GC2, GC3: GC contents of the first, second, third position of codon, respectively.
(TIF)

Figure S4 **Pipeline of the transcriptome and DGE bioinformatic analysis.** The Illumina reads with length of 75, 70, 65, 60, 55 and 50 bp were individually assembled using Edena, SOAPdenovo and Velvet, respectively. Contigs obtained from each set of reads by using every assembler with the optimized parameter [E75, E70, E65, E60, E55 and E50 assembled by Edena; S75, S70, S65, S60, S55 and S50 assembled by SOAPdenovo; V75, V70, V65, V60, V55 and V50 assembled by Velvet; and contigs provided by the commercial assembler service (CC)] were pooled and reassembled with CAP3. Final transcripts were evaluated, annotated and analyzed. For DGE analysis, DGE tags were filtered and clean tags were mapped to the final assembly. Differentially expressed transcripts (DETs) were screened by edgeR. Then, we used hypergeometric test to identify DETs, SETs and functionally enriched transcripts between each two samples.
(TIF)

Table S1 **Statistics of CAP3 reassembly output of sweet potato transcriptome.**
(XLS)

Table S2 **Validation of new genes by RT-PCR amplification and Sanger sequencing.**
(XLS)

Table S3 **Sequence annotations of sweet potato transcripts and the gene expression levels.**
(RAR)

Table S4 **Codon usage of sweet potato.**
(XLS)

Table S5 **Comparison of two assembly strategies on** ***de novo*** **assembly of sweet potato transcriptome.**
(XLS)
