# Supplementary figures and images for: Digital Gene Expression Analysis Based on Integrated De Novo Transcriptome Assembly of Sweet Potato [Ipomoea batatas (L.) Lam.]
Source: PLoS One. 2012 Apr 27;7(4):e36234. doi: 10.1371/journal.pone.0036234 (PMC3338685; doi:10.1371/journal.pone.0036234)

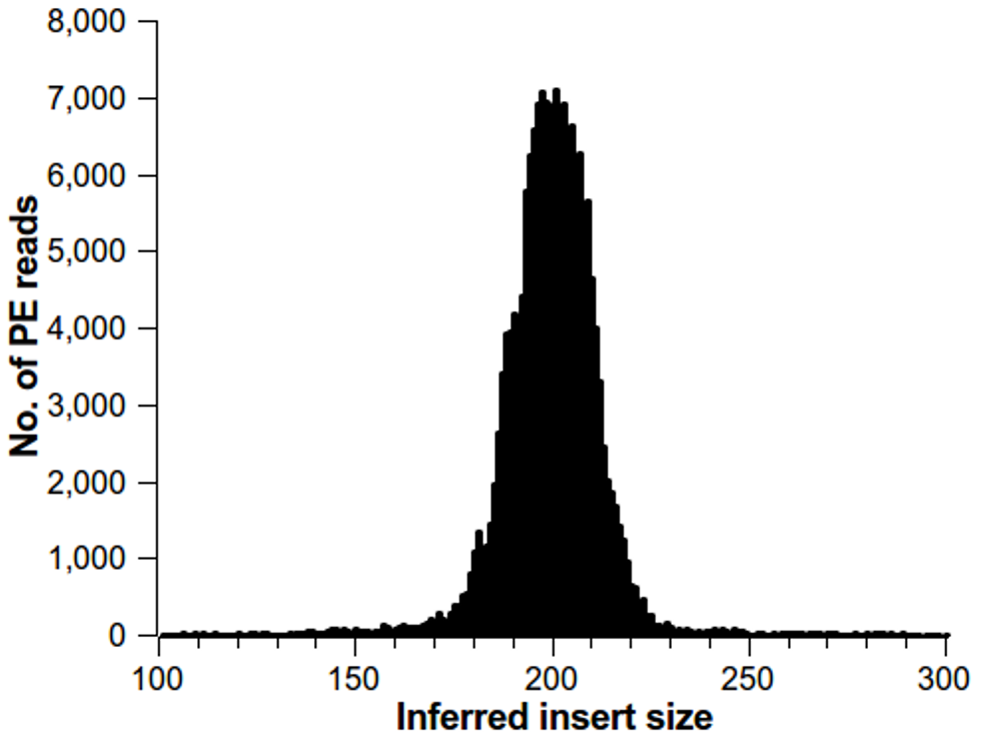

Supplement: Figure S1 — The insert size histogram of PE reads. The insert size of PE reads was inferred by mapping the PE reads to a chloroplast genome of I. purpurea (GenBank Accession Number NC_009808) which is a species close to I. batatas. (TIF) [file pone.0036234.s001.tif]

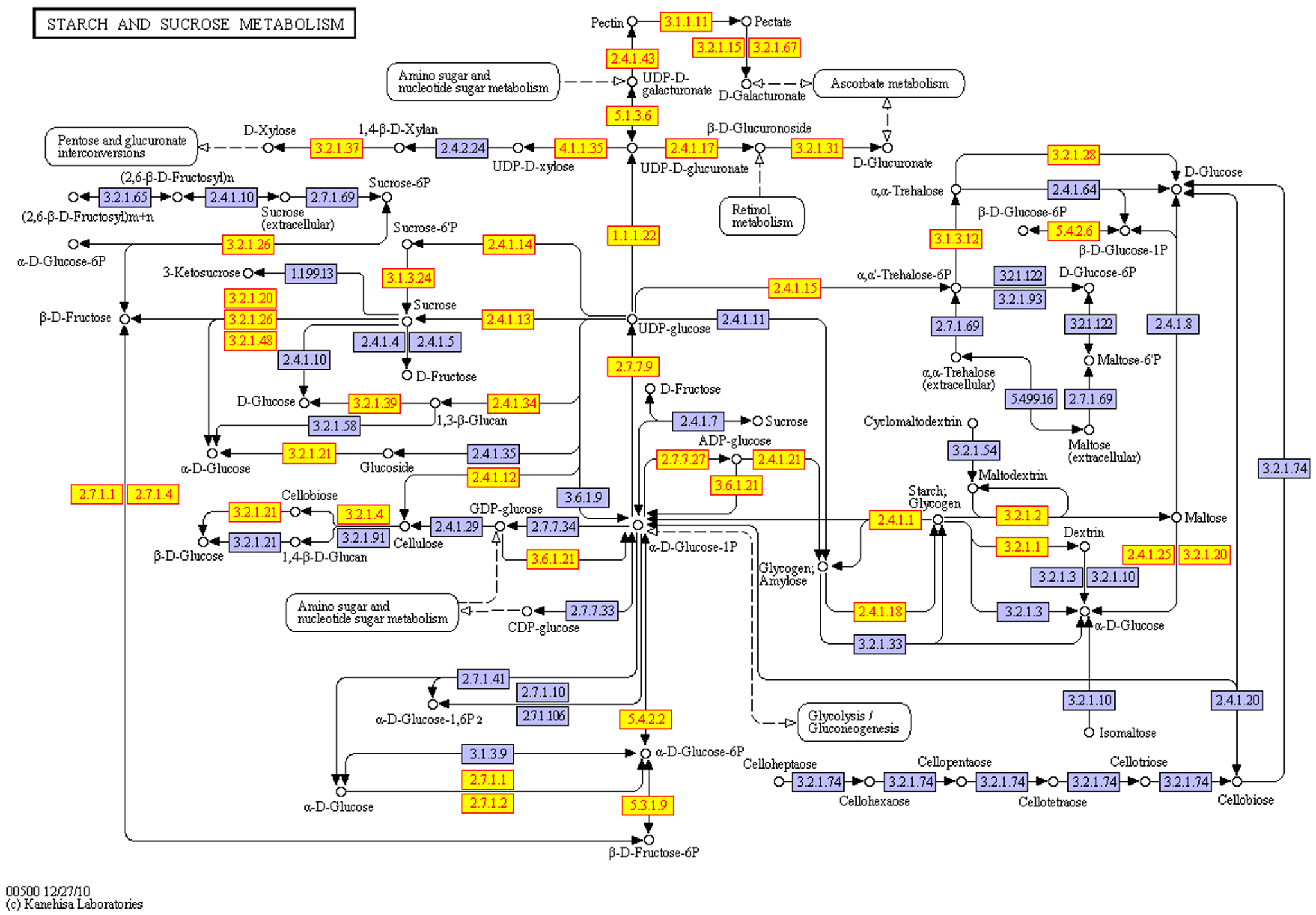

Supplement: Figure S2 — Map for KEGG starch and sucrose metabolism pathway of sweet potato. ECs in red were found in this study. (TIF) [file pone.0036234.s002.tif]

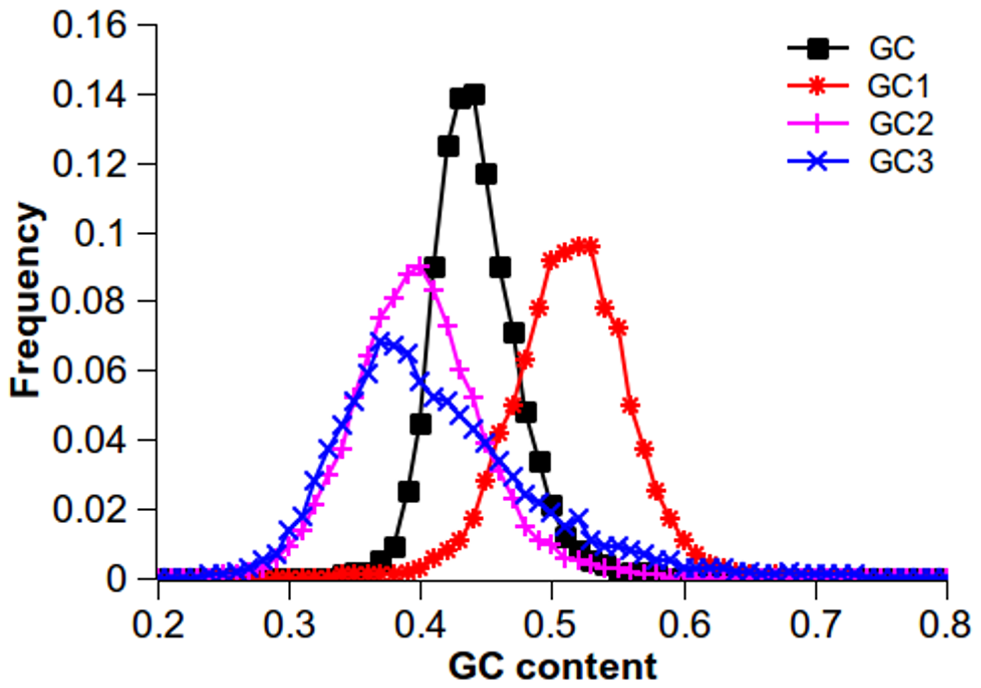

Supplement: Figure S3 — Distribution of GC in the coding region of sweet potato. 9,933 transcripts with ORF≥600 bp were used for GC content analysis. GC: GC contents of entire ORFs; GC1, GC2, GC3: GC contents of the first, second, third position of codon, respectively. (TIF) [file pone.0036234.s003.tif]

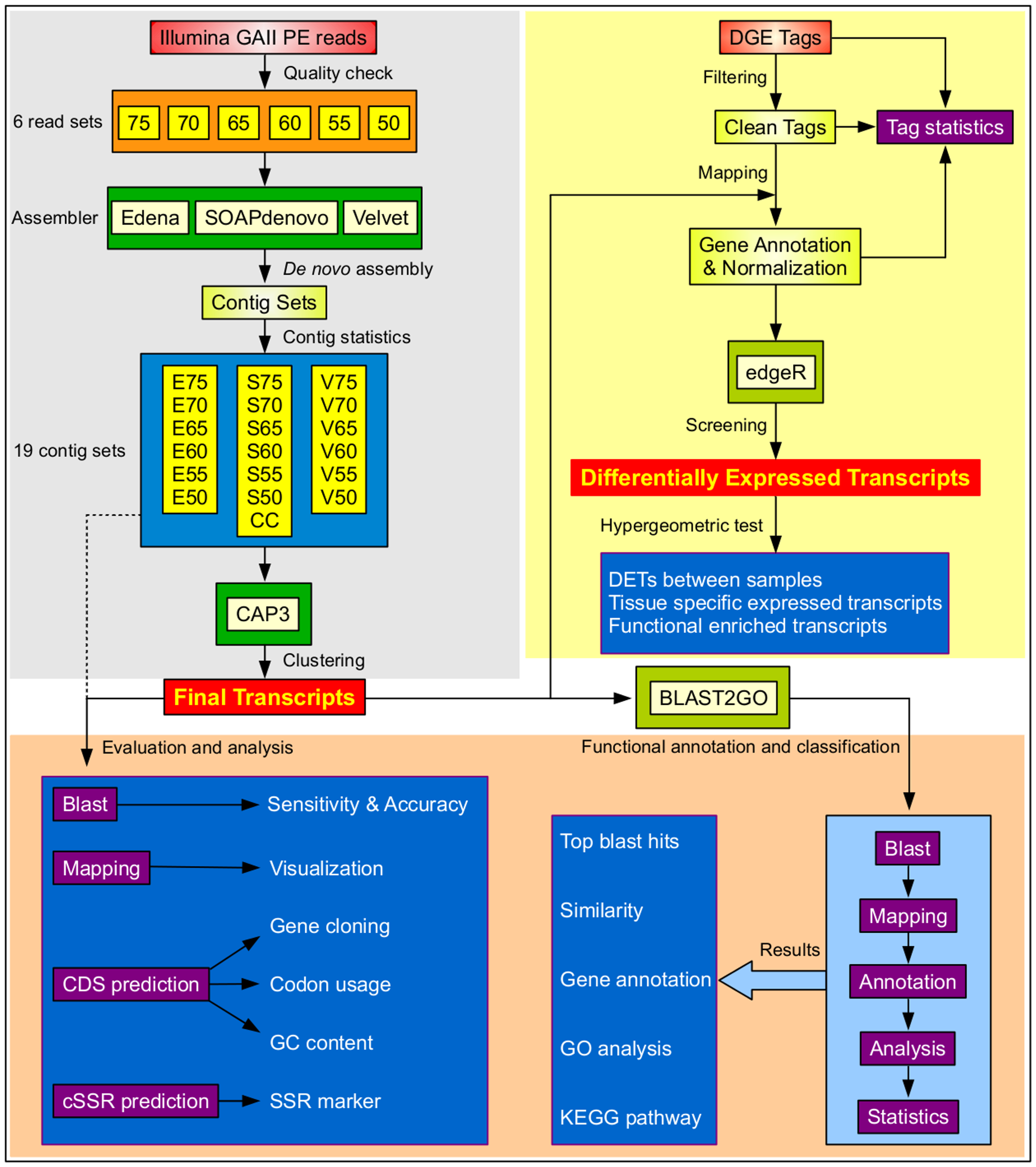

Supplement: Figure S4 — Pipeline of the transcriptome and DGE bioinformatic analysis. The Illumina reads with length of 75, 70, 65, 60, 55 and 50 bp were individually assembled using Edena, SOAPdenovo and Velvet, respectively. Contigs obtained from each set of reads by using every assembler with the optimized parameter [E75, E70, E65, E60, E55 and E50 assembled by Edena; S75, S70, S65, S60, S55 and S50 assembled by SOAPdenovo; V75, V70, V65, V60, V55 and V50 assembled by Velvet; and contigs provided by the commercial assembler service (CC)] were pooled and reassembled with CAP3. Final transcripts were evaluated, annotated and analyzed. For DGE analysis, DGE tags were filtered and clean tags were mapped to the final assembly. Differentially expressed transcripts (DETs) were screened by edgeR. Then, we used hypergeometric test to identify DETs, SETs and functionally enriched transcripts between each two samples. (TIF) [file pone.0036234.s004.tif]
